# T1 Glottic Carcinoma: Do Comorbidities, Facility Characteristics, and Sociodemographics Explain Survival Differences across Treatment Types?

**DOI:** 10.1177/0194599815572112

**Published:** 2015-02-24

**Authors:** Stephanie Misono, Schelomo Marmor, Bevan Yueh, Beth A. Virnig

**Affiliations:** 1 Department of Otolaryngology/Head and Neck Surgery, University of Minnesota, Minneapolis, Minnesota, USA; 2 Department of Surgery, University of Minnesota, Minneapolis, Minnesota, USA; 3 Division of Health Policy & Management, School of Public Health, University of Minnesota, Minneapolis, Minnesota, USA

**Keywords:** laryngeal cancer, glottic cancer, radiation, endoscopic laryngeal surgery, survival

## Abstract

**Objective:**

Recent large-scale studies have observed differences in survival following treatment for early laryngeal carcinoma depending on treatment type but were not able to take sociodemographic, comorbidity, and facility data into account. The objective of this study was to determine whether survival differences across treatment types persist when these factors are included in the analysis.

**Study Design:**

Retrospective cohort analysis.

**Setting:**

Linked Surveillance, Epidemiology, and End Results (SEER)–Medicare data files.

**Subjects and Methods:**

Medicare beneficiaries who were identified through the SEER registries (1991-2009) as having T1 glottic squamous cell carcinoma (scca) and a known treatment type were included.

**Results:**

A total of 2338 patients with incident T1 glottic scca were identified. Most were white and male. Treatment type was radiation only in 47%, local surgery and radiation in 39%, and local surgery only in 14%. Black race and increased comorbidities were associated with worse survival. When sociodemographics, comorbidities, and facility characteristics were taken into account, survival differences were observed across treatment types, with those receiving local surgery demonstrating better overall and cancer-specific survival.

**Conclusion:**

These results suggest that following treatment of T1 glottic scca, there may be survival differences across treatment types beyond those explained by sociodemographic, comorbidity, and facility characteristics.

Most laryngeal cancers are diagnosed in the early stages, and survival rates are generally excellent. Traditionally, single-modality treatment with either radiation or surgery (typically endoscopic surgery) has been recommended, and it is thought that oncologic results are comparable between the treatment approaches. However, more recently, some large-scale studies have observed differences in survival following treatment for early laryngeal carcinoma depending on treatment type.^1-3^ These studies, which included a study of the National Cancer Database and a population-based study using the Surveillance, Epidemiology, and End Results (SEER) cancer registries, observed better survival associated with the use of local surgery as part of treatment.

In some cases, the studies were limited by the ability to distinguish between T1 and T2 tumors and examined localized laryngeal cancer as a mixed group of T stages. Some were also limited by the inability to evaluate for the impact of factors such as income, hospital characteristics, and patient comorbidities on differential survival across treatment types. These factors are important to examine since they have all been shown to be associated with both treatment choice and survival.^1,4-8^ Some of these factors, particularly sociodemographics and hospital characteristics, might also influence evaluation of and clinical decision making for a patient who presents with laryngeal cancer, as could the use of technology such as laryngovideostroboscopy.

The objective of this study was to determine whether the previously observed survival difference across treatment types persisted after adjustment for sociodemographic characteristics, hospital characteristics, type of workup, and patient comorbidities.

The SEER-Medicare files were used to examine this issue because they have been shown to be a good source of information on cancer surgery^9^ and radiation^10^ and also allow examination of the additional patient and facility characteristics that we sought to consider. Based on prior findings, we hypothesized that better survival following treatment for T1 glottic carcinoma would be associated with treatment incorporating local surgery, even when sociodemographic characteristics, hospital characteristics, workup, and patient comorbidities were taken into account.

## Methods

### Data

We used the SEER cancer registry data linked to Medicare enrollment and utilization data (SEER-Medicare). The SEER cancer registries provide population-based cancer surveillance for 18 areas that represent approximately 28% of the United States. The SEER-Medicare Patient Entitlement and Diagnosis Summary File collects patient demographic and tumor characteristics, including age at diagnosis, race, primary tumor site, histology type, tumor stage, diagnostic confirmation, type of surgery, radiation, vital status, and cause of death (per death certificate).^11^

Medicare provides comprehensive health care for approximately 97% of the US population aged 65 years or older.^12^ Cancer cases reported to SEER have been matched to the Medicare master enrollment file to facilitate population-based health services research. Claims for hospitalizations and inpatient procedures are available in the Medicare Provider Analysis and Review (MedPAR) and National Claims History (NCH) files, and office visits are captured through a combination of NCH files for provider charges and outpatient standard analytical files for facility charges. In addition to the SEER data and Medicare claims, the National Cancer Institute maintains a hospital file of all providers in the SEER-Medicare data. The annual files include the number of Medicare-certified hospital beds and location (urban vs rural). Variables in the hospital file are abstracted from the Provider of Services and Healthcare Cost Report files, which are maintained by the Center for Medicare and Medicaid Services. This work was approved by the SEER-Medicare program as part of a data use application and was deemed exempt from review by the Institutional Review Board of the University of Minnesota as it used existing de-identified data.

### Patients

**Figure 1:**
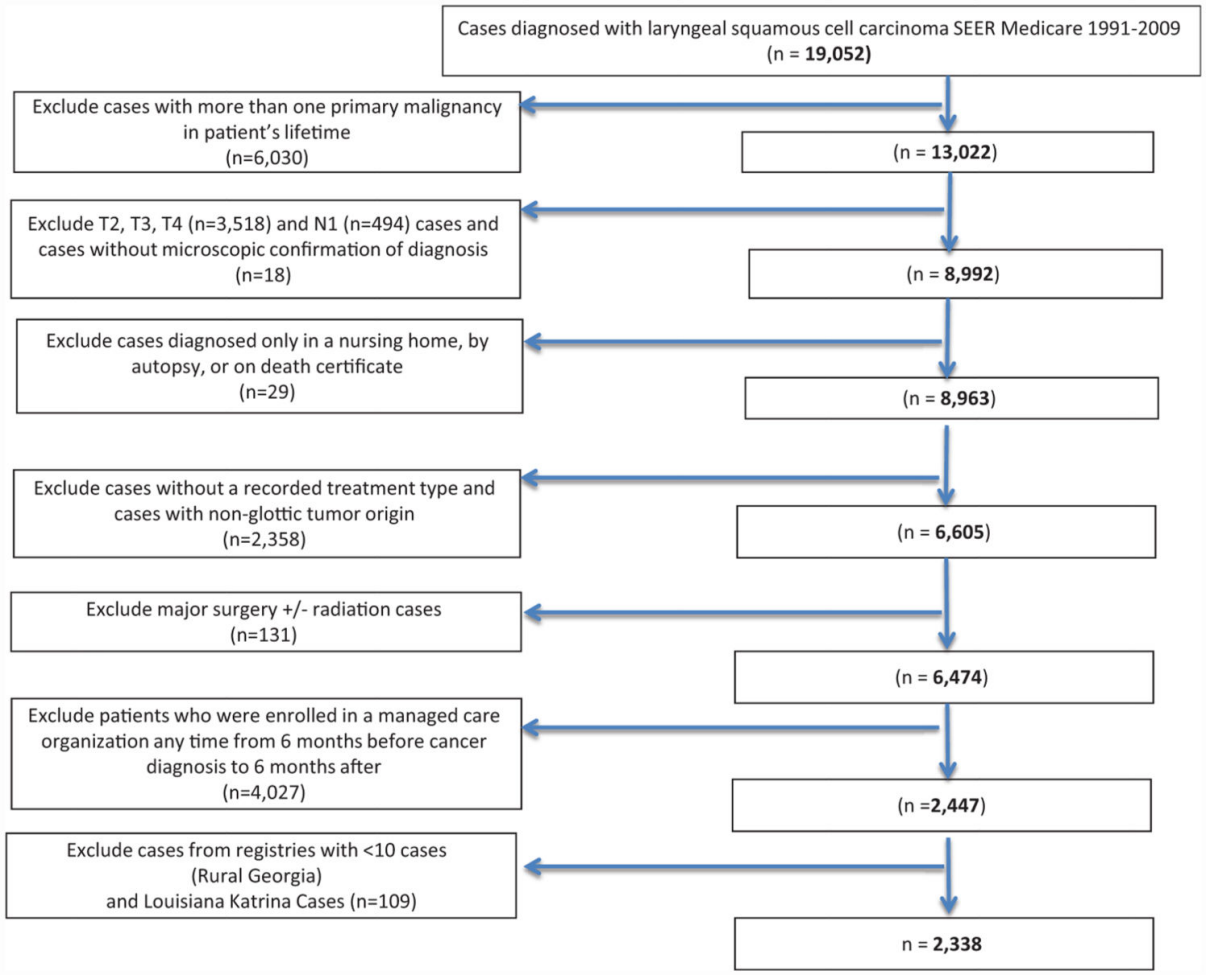
Cohort selection.

We limited inclusion to patients aged 66 years or older^9^ who had T1 laryngeal cancer from 1991 to 2009. T staging was available for the entire study period, allowing comparisons across time; analysis was also limited to patients whose cancer was either their only or first primary. Inclusion criteria included pathologically confirmed squamous cell carcinoma, and patients with N0 or N missing were included. The N missing was expected to predominantly represent N0 given the typical tendency of T1 laryngeal cancer to present without cervical metastasis.^13^ Excluded from the study were patients diagnosed only by autopsy, death certificate, or nursing home; patients with no recorded treatment; cases with nonglottic tumor origin; and patients treated with major surgery (30-80 in SEER coding, including partial laryngectomy, hemilaryngectomy, total/radical laryngectomy, and pharyngolaryngectomy). Analyses were restricted to Medicare Part A and B enrollees in the SEER-Medicare who were enrolled continuously in Medicare's fee-for-service program. Patients who were enrolled in a managed care organization any time from 6 months before cancer diagnosis to 6 months after diagnosis or death were excluded (because Medicare files do not include insurance claims data on managed care enrollees). Patients in registries with n < 10 cases (rural Georgia) and cases registered in Louisiana following Hurricane Katrina were also excluded.^14^ Stepwise ascertainment of our study cohort is represented in the accompanying diagram (Figure 1).

Treatment was categorized as radiation only, local surgery and radiation, and local surgery only. As in prior studies,^3^ surgical codes for local surgery included local tumor destruction and local tumor excision (10-28 in SEER coding). Radiation therapy included beam radiation and Radiation, NOS (not otherwise specified).

We hypothesized a priori that the patterns in the receipt of stroboscopy and laryngoscopy could affect treatment and survival for patients in our cohort, and therefore workup was also included in our proportional hazards models. We classified patients as having received stroboscopy (HCPCS CPT code 31579), flexible laryngoscopy (HCPCS CPT code 31575), or other (all other CPT workup codes such as direct laryngoscopy with biopsy) if we found any claims-based evidence of administration codes in the Medicare claims within a 90-day window before or after diagnosis.^15,16^ Based on previous studies in SEER-Medicare, we determined that 1 or more paid claims was sufficient evidence to determine that a patient had undergone a workup.^9,17^ We used a 90-day window prior to diagnosis to allow for delays due to coordinating efforts or postsurgical tests that might delay treatment initiation.

### Hospital Characteristics

To evaluate hospital-level factors associated with treatment and survival of T1 glottic carcinoma, we linked each patient to a single hospital during the study period using the SEER facility identifier associated with laryngeal cancer treatment. We categorized the number of hospital beds in quartiles (11-193, 194-337, 338-504, 505-1857, missing) and also identified the location (urban/rural) of the hospitals in which patients were treated.

### Statistical Analysis

We evaluated the unadjusted differences between demographic and hospital characteristics among patients in our cohort. We then used multivariate logistic regression to evaluate the factors associated with the receipt of stroboscopy, after adjusting for the case mix of patients within facilities. After assessing unadjusted relationships, we evaluated the association between treatment type and 5-year hazard of death using Kaplan-Meier methods and Cox proportional hazards modeling. All multivariate models used patients as the unit of analysis and were adjusted for age at diagnosis, race, sex, Charlson score (0, 1, ≥2), year of diagnosis, urban/rural designation, workup category (stroboscopy or laryngoscopy or other), patient income quartiles, and hospital bed quartiles. We performed sensitivity analyses examining the impact of restricting our Cox models to large registries (to ensure that small registries did not skew results), accounting for delayed billing by changing the time windows (30, 60, and 90 days after SEER date of diagnosis) of claims data coding for stroboscopy use and different ways to measure hospital volume (such as number of procedures). We did not identify any significant impact on study outcomes. These analyses did not produce results of different magnitude or direction.

All statistical analysis was completed using SAS software, version 9.3 (SAS Institute, Cary, North Carolina). All *P* values were 2-sided, with *P* < .05 considered significant. Our study was exempted from review by the Human Subjects Committee of the University of Minnesota's institutional review board because it used a preexisting de-identified data source.

## Results

### Description of Population

We identified 2338 patients with incident T1 laryngeal squamous cell carcinoma in the SEER registries from 1991 to 2009 (Table 1). Eighty-five percent were male and 15% were female. Eighty-five percent were non-Hispanic white, 7% were black, and 8% were other. Most patients were aged 66 to 74 years. Within our population, 55% had a Charlson comorbidity score of 0 and 45% had a score of 1 or greater. Most of our patients were treated in an urban setting. Income and hospital volume varied widely and are also presented in Table 1. Most patients received a workup code for laryngoscopy, and 7% underwent stroboscopy. Factors associated with the use of stroboscopy included later diagnosis year (2003-2009), upper quartile income, and treatment with local surgery only (data not shown, available upon request).

### Characteristics of Treatment

Among all patients with localized squamous cell carcinoma of the larynx diagnosed in 1991 to 2009, most were treated with radiation only (47%); 39% were treated with both local surgery and radiation and 14% with local surgery only.

### Unadjusted Relationship between Treatment Type and Survival

**Figure 2:**
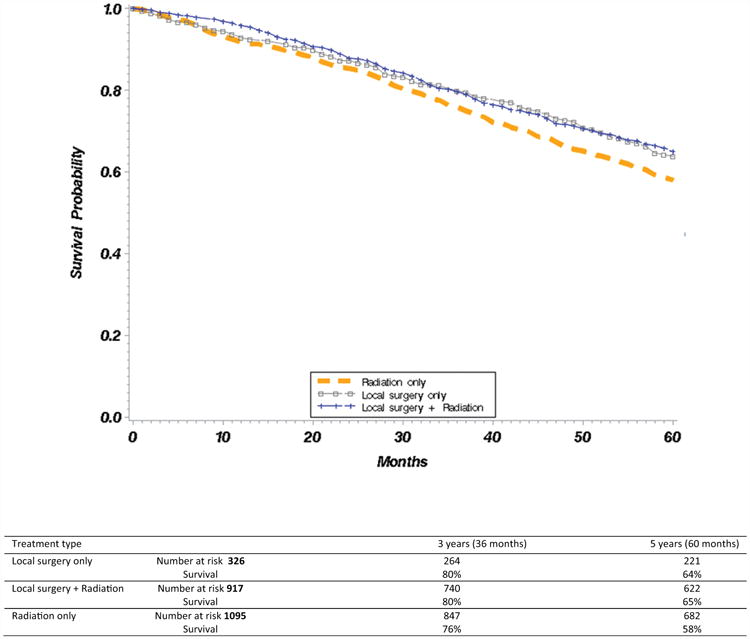
Kaplan-Meier curves of survival by treatment type. Overall survival, T1 glottic squamous cell carcinoma, Surveillance, Epidemiology, and End Results–Medicare; diagnosed 1991 to 2009.

**Figure 3:**
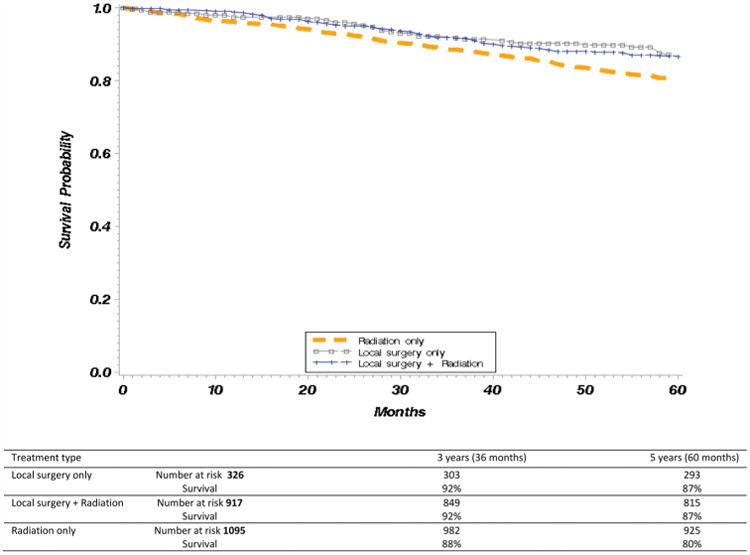
Kaplan-Meier curves of survival by treatment type. Cancer-specific survival, T1 glottic squamous cell carcinoma, Surveillance, Epidemiology, and End Results–Medicare; diagnosed 1991 to 2009.

Kaplan-Meier curves comparing overall survival between each of the treatment categories demonstrated differences within 3 years after treatment and persisted beyond 5 years after treatment (Figure 2). Similar Kaplan-Meier analyses were performed examining cancer-specific survival across treatment categories and demonstrated similar survival in patients who received local surgery with or without radiation, with worse survival in patients who received radiation only (Figure 3).

### Impact of Patient Factors and Treatment Type on Survival

We examined how sociodemographic and clinical factors influenced overall survival using Cox proportional hazard modeling (Table 2). When adjusted for gender, age, race, registry, Charlson comorbidity score, geography (urban/ rural), income quartiles, workup code, hospital size (bed quartiles), and treatment type, the hazard of death over the study period was essentially stable. The hazard of death was higher in blacks than in non-Hispanic whites and higher for patients with higher levels of comorbidity (Charlson score ≥2). Neither geographic location nor hospital characteristics had a meaningful impact on hazard of death. The relative hazard of death was significantly lower in patients whose treatment included local surgery, with the analysis adjusted for the other factors described above.

## Discussion

In this examination of patients with T1 glottic squamous cell carcinoma identified in SEER-Medicare data spanning 1991 to 2009, we observed better survival among patients whose treatment included local surgery. Worse survival was associated with black race and greater medical comorbidity. No cancer-specific survival differences were observed with the year of diagnosis, age, gender, urban/rural location, income, workup, or hospital volume. Better survival was associated with the inclusion of local surgery in the treatment type, adjusting for all of the factors above.

Prior literature had suggested that treatment type might influence survival following treatment of laryngeal cancer,^1,2^ but relatively little is known about this phenomenon in early laryngeal cancer. In our prior work, we had observed that treatment including local surgery appeared to be associated with better survival,^3^ but with SEER data alone, we were unable to assess the potential impact of other factors that could independently affect treatment type and/or survival. Therefore, in this study, we aimed to determine whether factors other than treatment type might account for the survival differences observed in prior studies. Our findings were consistent with prior observations that nonwhite race^18,19^ and greater medical comorbidities^20^ are associated with worse survival. However, both overall and cancer-specific survival differences were associated with treatment type incorporating local surgery even when those factors were taken into account.

We observed relatively infrequent use of laryngovideostroboscopy in the evaluation of these patients, despite literature suggesting that it may be a useful technology in this setting.^21-23^ This may be because the equipment is expensive and may not be readily available in many practices. We did note that use of laryngovideostroboscopy in the workup was associated with a treatment type that included local surgery and higher income and was more common for persons more recently diagnosed. No survival difference was detected. These observations should be interpreted with caution given the small number of patients represented in this group, and further work in this area is needed before clinical significance can be interpreted. Similarly, facility characteristics, although previously observed to be associated with differences in survival,^1^ did not appear to influence survival in this analysis. This may perhaps be because subspecialty treatment may be collinear with facility volume.

Strengths of this study include the large number of patients for whom data were available, far exceeding what might be assessable in a single or small multi-institutional study, and the ability to extend prior analyses with information on medical comorbidities as well as sociodemographic factors that may have had independent associations with differences in survival. We were able to focus the present analysis on patients who had T1 stage disease of the glottic subsite, whereas our prior study included all localized laryngeal cancers because of the lack of availability of T-stage data for most of the included patients. The additional precision in this study allowed us to decrease variability in cancer-related survival expectations at the time of diagnosis.

Limitations of the study include those common to all analyses of administrative data, which include potential mis-classification or other errors that may influence findings in ways that cannot be independently assessed. We were somewhat surprised to note that a larger proportion of these T1 stage patients (39%) were treated with local surgery and radiation than we might have expected based on our prior study (25% of cohort); this remained true when SEER treatment codes were compared with Medicare billing codes. We performed sensitivity analyses examining the impact of changing time windows as well as different ways to represent hospital volume (such as number of procedures) and did not identify any major impact on study outcomes. We also assessed whether age and comorbidity were differentially associated with treatment type, perhaps accounting for some of the survival differences we observed, but no such association was detected. Another potential limitation is generalizability, since the population included in this analysis is predominantly elderly; however, since most patients diagnosed with early laryngeal cancer are aged 60 or older,^3,24^ our findings are likely to be relevant for many patients with the disease. An additional potential limitation of generalizability is the exclusion of patients who enrolled in managed care organizations. Although necessary, this reduced the total number of patients eligible for inclusion in the study. As in prior work, we are unable to assess for possible patterns in clinical decision making that may have influenced treatment type and survival outcomes.

These findings underscore the need for further study in this area so that patients can receive nuanced counseling as part of treatment decision making. We did not observe any significant relationships between center characteristics and survival. Prospective randomized studies, while challenging to implement, are needed to reach greater clarity with respect to these survival differences that persist even when a wide variety of patient and disease characteristics are taken into account. Our results illustrate that treatment decision making will need to be carefully considered for each individual patient with the knowledge that our long-held assumptions about survival equivalence across treatments for early laryngeal cancer may need reconsideration.

## Conclusions

In this study of patients with T1 glottic carcinoma identified through SEER and enrolled in Medicare, we observed that worse survival was associated with black race and greater medical comorbidity. We also observed that overall and cancer-specific survival were better among patients whose treatment included local surgery, independent of sociodemographic and clinical factors such as comorbidity and hospital volume. These findings suggest a need for further inquiry into factors associated with treatment decision making and survival differences in this patient population.

## Figures and Tables

**Table 1:** Description of Population, Localized Squamous Cell Carcinoma, 1991-2009 (N = 2338).

Demographic or Treatment Characteristic	n	%
Gender		
Male	1989	85
Female	349	15
Race		
White	1990	85
Black	168	7
Other	180	8
Age, y		
66-69	532	23
70-74	690	29
75-79	580	25
80-84	345	15
≥85	191	8
Diagnosis year		
1991-1996	667	28
1997-2002	789	34
2003-2009	882	38
Urban/rural		
Urban	2082	89
Rural	256	11
Income quartile		
Q1 ($8132-$34,011)	459	20
Q2 ($34,012-$44,490)	459	20
Q3 ($44,491-$59,507)	460	20
Q4 ($59,508-$200,008)	430	18
Missing	530	22
Workup category		
31579	157	7
31575	1410	60
Other	771	33
Hospital beds		
11-193	539	23
194-337	542	23
338-504	536	23
505-1857	549	24
Missing	172	7
Charlson comorbidity score		
0	1285	55
1	644	28
≥2	409	17
Treatment type		
Radiation	1095	47
Local only	326	14
Local surgery ± radiation	917	39

**Table 2:** Factors Associated with 5-Year Relative Hazard of Death, Cox Proportional Hazard Models.^a^^,^^b^

Characteristic	Hazard Ratio [95% Confidence Interval]	P
Treatment group		
Radiation only	Referent	
Local only	**0.64 [0.42, 0.97]**	**.04**
Local and radiation	**0.71 [0.54, 0.93]**	**.01**
Year of diagnosis		
1995-1999	Referent	
2000-2004	0.81 [0.52, 1.28]	.37
2005-2009	0.89 [0.56, 1.41]	.62
Gender		
Male	Referent	
Female	0.97 [0.69, 1.38]	.88
Race		
Non-Hispanic white	Referent	
Black	**1.68 [1.12, 2.49]**	**.01**
Other or unknown	1.32 [0.86, 2.02]	.21
Age, y		
66-69	Referent	
70-74	0.84 [0.60, 1.19]	.34
75-79	0.95 [0.66, 1.35]	.76
80-84	1.31 [0.89, 1.94]	.18
≥85	1.42 [0.89, 2.25]	.14
Urban/rural		
Urban	Referent	
Rural	1.26 [0.85, 1.88]	.25
Income quartiles		
Q1 ($8132-$34,011)	Referent	
Q2 ($34,012-$44,490)	0.99 [0.70, 1.42]	.98
Q3 ($44,491-$59,507)	0.78 [0.52, 1.16]	.22
Q4 ($59,508-$200,008)	0.68 [0.44, 1.05]	.08
Missing	0.71 [0.42, 1.21]	.20
Workup		
Laryngoscopy	Referent	
Stroboscopy	1.04 [0.78, 1.37]	.81
Other	0.93 [0.54, 1.60]	.80
Hospital bed quartiles		
11-193	Referent	
194-337	0.89 [0.62, 1.27]	.53
338-504	0.77 [0.53, 1.12]	.17
505-1857	0.96 [0.67, 1.36]	.80
Missing	1.13 [0.68, 1.87]	.64
Charlson comorbidity score		
0	Referent	
1	1.12 [0.83, 1.51]	.44
≥2	**1.56 [1.13, 2.15]**	**.01**

a Adjusted for age at diagnosis, race, sex, Charlson score, year of diagnosis, urban/rural designation, workup category, patient income quartiles, hospital bed quartiles, and treatment type.

b Bold denotes *P* < .05.
